# Activation of violaxanthin cycle in darkness is a common response to different abiotic stresses: a case study in *Pelvetia canaliculata*

**DOI:** 10.1186/1471-2229-11-181

**Published:** 2011-12-26

**Authors:** Beatriz Fernández-Marín, Fátima Míguez, José María Becerril, José Ignacio García-Plazaola

**Affiliations:** 1 Department of Plant Physiology and Ecology, University of the Basque Country (UPV/EHU), Apdo 644, 48080 Bilbao, Spain

## Abstract

**Background:**

In the violaxanthin (V) cycle, V is de-epoxidized to zeaxanthin (Z) when strong light or light combined with other stressors lead to an overexcitation of photosystems. However, plants can also suffer stress in darkness and recent reports have shown that dehydration triggers V-de-epoxidation in the absence of light. In this study, we used the highly stress-tolerant brown alga *Pelvetia canaliculata *as a model organism, due to its lack of lutein and its non-photochemical quenching independent of the transthylakoidal-ΔpH, to study the triggering of the V-cycle in darkness induced by abiotic stressors.

**Results:**

We have shown that besides desiccation, other factors such as immersion, anoxia and high temperature also induced V-de-epoxidation in darkness. This process was reversible once the treatments had ceased (with the exception of heat, which caused lethal damage). Irrespective of the stressor applied, the resulting de-epoxidised xanthophylls correlated with a decrease in Fv/Fm, suggesting a common function in the down-regulation of photosynthetical efficiency. The implication of the redox-state of the plastoquinone-pool and of the differential activity of V-cycle enzymes on V-de-epoxidation in darkness was also examined. Current results suggest that both violaxanthin de-epoxidase (VDE) and zeaxanthin-epoxidase (ZE) have a basal constitutive activity even in darkness, being ZE inhibited under stress. This inhibition leads to Z accumulation.

**Conclusion:**

This study demonstrates that V-cycle activity is triggered by several abiotic stressors even when they occur in an absolute absence of light, leading to a decrease in Fv/Fm. This finding provides new insights into an understanding of the regulation mechanism of the V-cycle and of its ecophysiological roles.

## Background

The violaxanthin (V) cycle, described in plants and green and brown algae (Müller et al. 2001), consists of the light-driven de-epoxidation of V to form antheraxanthin (A) and then zeaxanthin (Z), and the epoxidation of Z back to V in darkness. The first reaction is catalyzed by violaxanthin deepoxidase (VDE), an enzyme that, for its activation, requires the acidic pH that results from photosynthetic proton pumping [1]. De-epoxidation of V to A + Z enhances and modulates the rate of thermal dissipation of the excess of excitation energy [2]. The biophysical mechanism by which this process occurs is still a matter of debate [3-7]. This process can be monitored by the decrease in chlorophyll *a *fluorescence yield, the so-called non-photochemical quenching (NPQ). Although light has been considered to be a requisite for the activation of VDE in all organisms studied until now, studies performed with non-natural systems have suggested that artificial induction of V de-epoxidation in darkness may be possible. In isolated thylakoids of lettuce, the dark-induction of the Z-dependent quenching of chlorophyll fluorescence mediated by external ATP supplies has been shown [8]. In *Arabidopsis *mutants lacking a chloroplast NAD Kinase, an accumulation of high levels of Z was observed in darkness [9]. More recently, Z accumulation in darkness has been observed in natural, cold-acclimated oak leaves [10] while several papers have confirmed that the de-epoxidation of V can be induced under natural conditions by desiccation [11,12], irrespective of the illumination of photosynthetic tissues.

In green algae and plants, apart from the presence of A + Z, a trans-thylakoidal proton gradient (ΔpH) is required to obtain the highest NPQ [2]. In brown algae, however, the generation of NPQ depends exclusively on the operation of the V-cycle, whereas acidic pH is only required to activate VDE [13]. The direct dependence of NPQ on Z and the lack of lutein, another carotenoid that contributes to NPQ [14], greatly facilitate the study of the functions and operation of the V-cycle, becoming brown algae excellent model species in such studies.

Among brown seaweeds, *Pelvetia canaliculata *is one of the most stress-tolerant species. It forms the highest band on the shore above *Fucus spiralis *and is very desiccation-tolerant, surviving for more than 7 days out of water [15], when it can lose up to 96% of its water content [16]. The unique desiccation tolerance ability of this species seems to rely on a symbiosis with the endophytic fungus: *Mycosphaerella acophylli *(Ascomycetes) [17]. But besides dehydration, the emersion of intertidal algae is associated with other stressors such as high temperatures that can damage their tissues [18]. Strong light is also a stress factor that intertidal algae have to deal with. A very recent paper has reported that *P. canaliculata *acclimates very quickly to high radiations based on an efficient carotenoids composition [19]. Furthermore, the high V-cycle pool size of *P. canaliculata *seems to be associated with its strong tolerance to abiotic stresses [20].

Since an increasing number of evidences support the dark operation of V-cycle under some environmental conditions, but its physiological role and regulation remains unknown, *P. canaliculata *has been used as a model species in this paper to provide further insights into this mechanism. Specifically, we have determined whether environmental factors, other than light, may trigger the activation of the V-cycle. Three environmental factors that this highly stress-tolerant species can eventually encounter during its lifespan have been studied in the absence of light: desiccation that occurs when thalli are exposed to air for a long time, and supraoptimal temperature and anoxia that occur at low tide when oxygen is depleted from the small intertidal pools. The second aim of this work was to verify whether the pool of A + Z, formed after VDE activation in darkness, is involved in the regulation of photochemical efficiency in the same way as the pool of A + Z, generated by high irradiance. This paper not only aims to gain an understanding of this protective mechanism, but also to look more deeply into the functioning and environmental role of the V-cycle.

## Methods

### Plant material

*Pelvetia canaliculata *(L.) Decn. et Thur. (also called channelled wrack) is a brown seaweed of the Fucaceae family distributed on the cold shores of the northern hemisphere. Adult thalli were collected from the Northern coast of Spain. Before any analysis, samples were covered with a tissue soaked in seawater and kept in darkness at room temperature and 100% Relative Humidity (RH), during 12 h, to allow V-cycle relaxation. For all the experiments, the last apical bifurcation of the thalli was used, since this is the most active part and that which is most directly exposed to desiccation.

### Experimental treatments

The thalli collected as previously described were exposed to different experimental treatments, as follows:

#### • Illumination

Samples over a tissue moistened with seawater were directly exposed to sunlight (Photon Flux Density -PFD- of 1500 µmol m^-2 ^s^-1^), for 15 or 60 min, and continuously remoistened with seawater to avoid desiccation. After light treatment, the thalli were maintained wet in darkness (to allow V-cycle re-epoxidation) for another 60 min.

#### • Desiccation

Samples were incubated in the dark for 24 h in glass hermetic chambers, containing a NaCl saturated solution (75% Relative Humidity -RH-). Rehydration was begun by directly spilling seawater over the thalli. Samples were then kept over a tissue wet with seawater and at 100% RH to allow recovery.

#### • Desiccation of pre-illuminated thalli

Bifurcated thalli, of which one of the branches was covered with aluminium foil, were exposed to sunlight for 15 min and then put into a hermetic chamber to start desiccation treatment as previously described.

#### • High temperature

Thalli were incubated in darkness at 32°C during 23 h, and then transferred to 17°C for 29 h further as recovery. To avoid desiccation, thalli were placed over a tissue wet with seawater and in a controlled atmosphere at 100% RH. As a control a second set of samples was maintained in darkness at 17°C and 100% RH.

#### • Immersion

Thalli were submerged in darkness in seawater in 2 mL volume vials for 10 and 24 h.

#### • Anaerobiosis

Thalli were incubated in darkness during 1, 6 and 17 h, in closed vacutainers in which the air was replaced by N_2 _gas. For controls, some samples were kept in vacutainers containing air. After the treatment, the samples were incubated in open vacutainers during 4 h to allow reoxygenation of tissues. During the incubations, all samples remained covered with a seawater-moistened tissue to avoid dehydration.

### Metabolic inhibitors

Since the acidification of lumen generated by the proton pumping of the ETC carriers in parallel to electron transport is required for the de-epoxidation of V, a set of inhibitors affecting either the activity of V-cycle enzymes, the transthylakoidal pH or the redox-state of the PQ pool, were infiltrated in samples that were then desiccated in darkness (as described above). Thalli were infiltrated with different concentrations of metabolic inhibitors dissolved in seawater with 1% ethanol to facilitate the dilution. For the infiltration, thalli were placed into vacutainers containing 5 mL of inhibitor solutions. By removing the air with a 50 mL syringe 5 times, a vacuum was created. After infiltration, samples were incubated into the solution for 1 h. Controls were treated with seawater and 1% ethanol following the same infiltration protocol. After inhibitor treatments, samples were either desiccated as described above or maintained hydrated. The following inhibitors (all from Sigma-Aldrich, Spain) were used:

#### • Dithiothreitol (DTT)

inhibits the enzyme VDE [21]. It was applied at a concentration of 5 mM.

#### • Salicyl-adoxime (SA)

inhibits the ZE activity [22]. It was applied at a concentration of 5 mM.

#### • Ammonium chloride (NH_4_Cl)

uncouples the transthylakoidal-ΔpH [13] by sequestering H + in the lumen. It was applied at a concentration of 100 mM.

#### • 2,5-Dibromo-6-isopropyl-3-methyl-1,4-benzoquinone (DBMIB)

avoids the oxidation of PQ by residing at the Q_0 _site of the cytochrome b6f complex [23]. It was applied at a concentration of 25 µM.

#### • Dichlorophenyl dimethylurea (DCMU)

avoids the reduction of PQ by blocking the electron flow from PS II [24]. It was applied at a concentration of 10 µM.

#### • n-Propyl-gallate (n-PG)

blocks the chlororespiratory oxidation of PQ mediated by the enzyme plastid terminal oxidase (PTOX) [25]. It was applied at a concentration of 1 mM.

#### • Far red (FR)

a modulated light of *λ *>700 nm provided by an halogen lamp with a FR filter was applied during all the desiccation of FR-treated thalli to maintain the PQ oxidized due to the induction of the PS I.

### Chlorophyll *a *fluorescence analysis

Fluorescence of chlorophyll *a *was measured with an imaging fluorometer (Handy FluorCam, P.S.I., Brno, Czech Republic, http://www.psi.cz), as described in [26]. Images of photochemical efficiency were captured before and immediately after each treatment in the thalli that were previously dark-adapted for at least 5 min. Fluorescence was detected by a high-sensitivity charge coupled device camera that produced images of 12-bit resolution. The instrument is driven by the FluorCam software package (FluorCam 7). First, images of the dark-adapted fluorescence level, F_0_, were determined using non-actinic measuring flashes provided by super-bright light emitting diodes (LEDs). Next, an 800 ms duration pulse of saturating light radiation (2000 µmol photon m^-2 ^s^-1^) generated by a halogen lamp was given to measure the maximum fluorescence level, Fm. The Maximal Photochemical Efficiency of Photosystem II (Fv/Fm) was calculated as (Fm-F_0_)/Fm. Pixel value images of the Fv/Fm were displayed as a false colour code ranging from blue (0.1) through green and yellow to red (0.9). The Fv/Fm was calculated for each pixel and then averaged for the total area of each sample. The NPQ was not measured because volume contractions associated with desiccation impedes the obtention of comparable Fm (reference Fm') and Fm' (actual Fm'). Thus, the decrease in Fv/Fm associated with sustained thermal dissipation was used as a proxy of NPQ.

### Chromatographic analysis

For pigment analysis, samples were immediately frozen in liquid N_2 _and stored at -80°C until use. Frozen samples were homogenized with a Tissue Tearor Homogenizer (Model 395, Dremel, Mexico) in pure acetone solution buffered with CaCO_3_. The extracts were centrifuged at 16100 g for 20 min, and supernatants were filtered with 0.2 mm PTFE filters (Teknokroma, Spain). Pigment separation was performed by HPLC with a reverse phase C18 column (Waters Spherisorb ODS1, 4.6 × 250 mm, Massachusetts, USA), following the method of [27], with the modifications described in [28]. During processing in the HPLC, samples were maintained at 4ºC in a refrigerated compartment. Identification and quantification was carried out with a photodiode array (PDA) detector. Retention times and conversion factors for pigments were the same as described by [27,28] The relative de-epoxidation state of the xanthophyll cycle pigments was estimated by the ratio (A + Z)/(V + A + Z) and represented by the abbreviation AZ/VAZ.

### Statistics

A one-way ANOVA was used to test for differences in AZ/VAZ and Fv/Fm values in response to light, desiccation, immersion and anoxia treatments, after Chochran's test to check for the homogeneity of variances. A Duncan post-hoc test was performed to discriminate among different times and treatments. When necessary, data were log-transformed. The Mann-Whitney *U *test was used for non-normal data. A linear regression was used to analyse the relationship between AZ/VAZ and Fv/Fm. Calculated *p*-values, coefficients and regression lines are indicated on the figures whenever significant at α = 0.05. All analyses were performed using the SPSS 17.0 statistical package.

## Results

### V-cycle operation upon illumination in *Pelvetia canaliculata*

**Figure 1:**
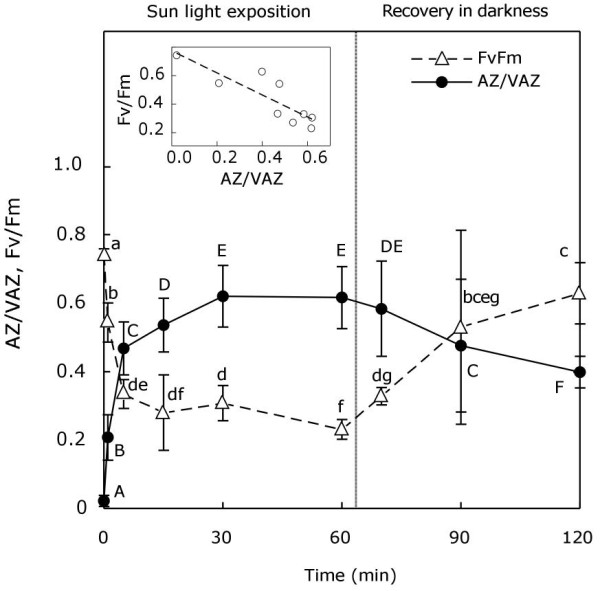
**De-epoxidation index of V-cycle (close cyrcles) and variations in Fv/Fm (open triangles) during light-darkness treatment**. Each point represents the mean ± SE (n = 5). Capital letters above circles show significant differences in the AZ/VAZ among times (*P *< 0.05). Small letters above triangles represent significant differences in the Fv/Fm among times (*P *< 0.05). Correlation between AZ/VAZ and Fv/Fm is shown in the inset (Pearson's correlation coefficient; *r *= 0.74).

The usual operation of the violaxanthin cycle (V-cycle) induced by light was ascertained for *P. canaliculata*. When exposed to sunlight, violaxanthin (V) was quickly de-epoxidised to antheraxanthin (A) and zeaxanthin (Z), this being 60% the fraction of the VAZ pool converted to A + Z in 30 min (Figure 1). During the exposure to darkness, Z and A were slowly re-epoxidised back to V, closing the cycle. Throughout the entire process, Fv/Fm followed an inverse pattern to AZ/VAZ, reaching the maximum value when AZ/VAZ was almost zero. Both parameters (Fv/Fm and AZ/VAZ) were negatively correlated (see insert in Figure 1).

### V-cycle and dehydration

**Figure 2:**
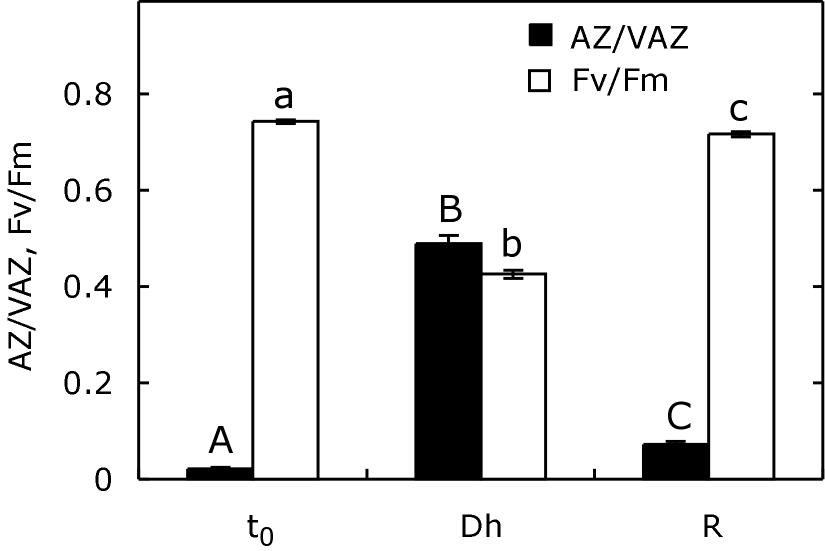
**De-epoxidation index of V-cycle and variations in Fv/Fm during a dehydration-rehydration treatment in darkness**. Dehydration (Dh) was performed at 75% RH for 24 h. V-cycle de-epoxidation index is represented by closed bars and Fv/Fm by open bars. Each bar represents the mean ± SE (n = 5). Letters above bars indicate significant differences for AZ/VAZ (A, B and C) and for Fv/Fm (a, b, c) along treatment (*P *< 0.05).

After verifying its regular activity under light, the V-cycle operation of *P. canaliculata *was analyzed in the absence of light during a dehydration-rehydration treatment (Figure 2), as described in the materials and methods section. During dehydration, and despite the darkness, V was de-epoxidised into A + Z, and the reverse reaction occurred after rehydration with seawater. As observed during the light experiment (Figure 1), the lowest Fv/Fm value was concomitant with the highest AZ/VAZ level (Figure 2).

**Figure 3:**
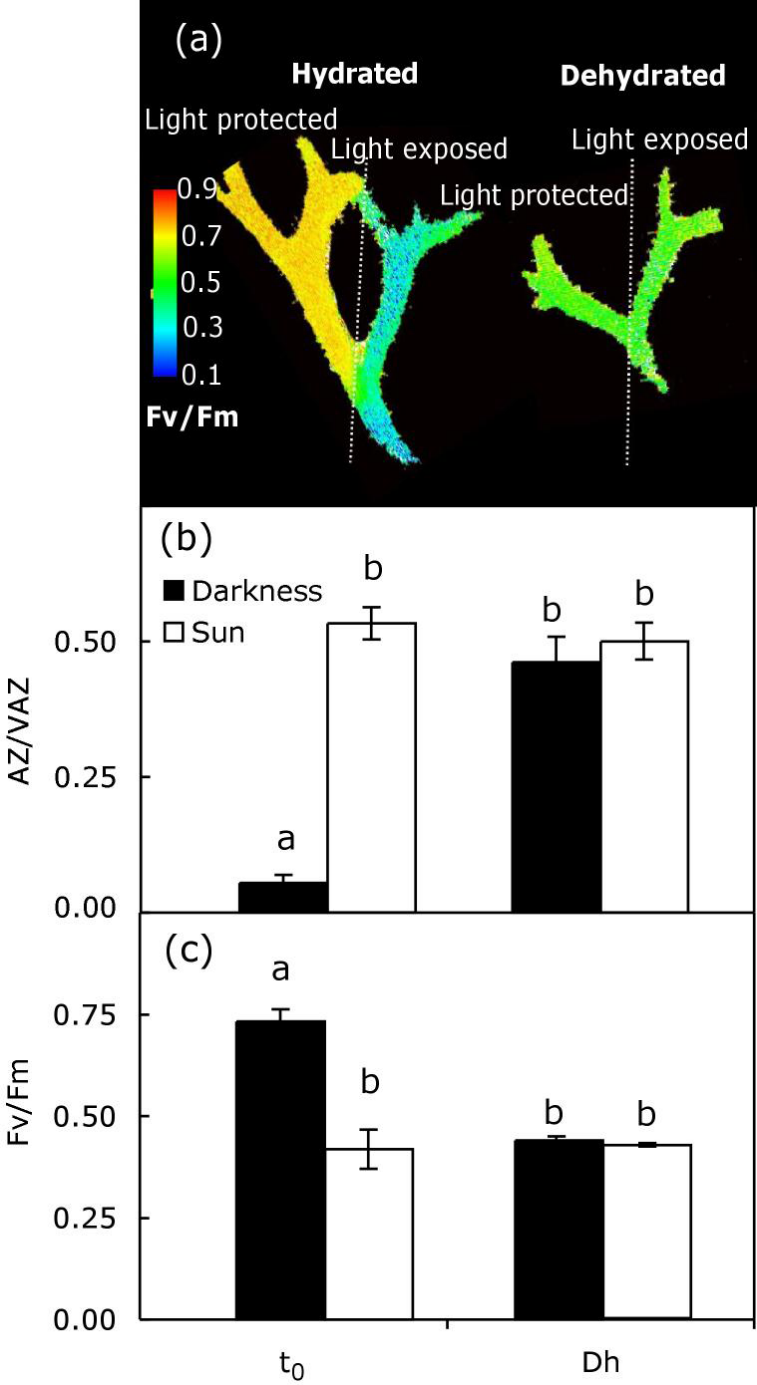
**De-epoxidation index of V-cycle and Fv/Fm variations during dark-dehydration after light or darkness pre-treatment**. In (**a**) it is shown a fluorescence imaging of thalli before (left) and after dehydration (right). Before dehydration, the right branch of each thallus had been exposed to sun, and the left one had been maintained in darkness. The de-epoxidation index of the V-cycle (**b**) and Fv/Fm (**c**) are shown before (t_0_) and after dark-dehydration (Dh) of pre-darkened (closed bars) and pre-illuminated samples (open bars). Each bar represents the mean ± SE (n = 5). Letters above bars indicate significant differences among treatments (*P *< 0.05).

To verify whether A + Z produced during dehydration may have different roles than that synthesised under light conditions, a new test was performed to compare their influence on Fv/Fm. For this purpose, samples of *P. canaliculata *were exposed to sunlight for 15 minutes, while half of each thallus was protected from light (with aluminium foil). After this light/darkness pre-treatment, all samples were dehydrated in the absence of light, as described previously. The illumination of the thalli increased the AZ/VAZ ratio to 0.53 ± 0.03. After dehydration, this index remained constant (0.50 ± 0.03) in pre-illuminated samples (Figure 3b, open bars) and increased to the same extent (0.46 ± 0.05) in non pre-illuminated thalli (Figure 3b, close bars). It should be pointed out that this A + Z was induced by light in the former, whereas it was generated in darkness during dehydration in the latter. The Fv/Fm reflected the changes on the de-epoxidation index of the V-cycle (Figure 3c): at the beginning of the experiment, Fv/Fm was significantly higher in dark-adapted samples than in pre-illuminated thalli, but during dehydration it decreased considerably, reaching the same value as in pre-illuminated thalli (Figure 3a,c).

### Supraoptimal temperature

**Figure 4:**
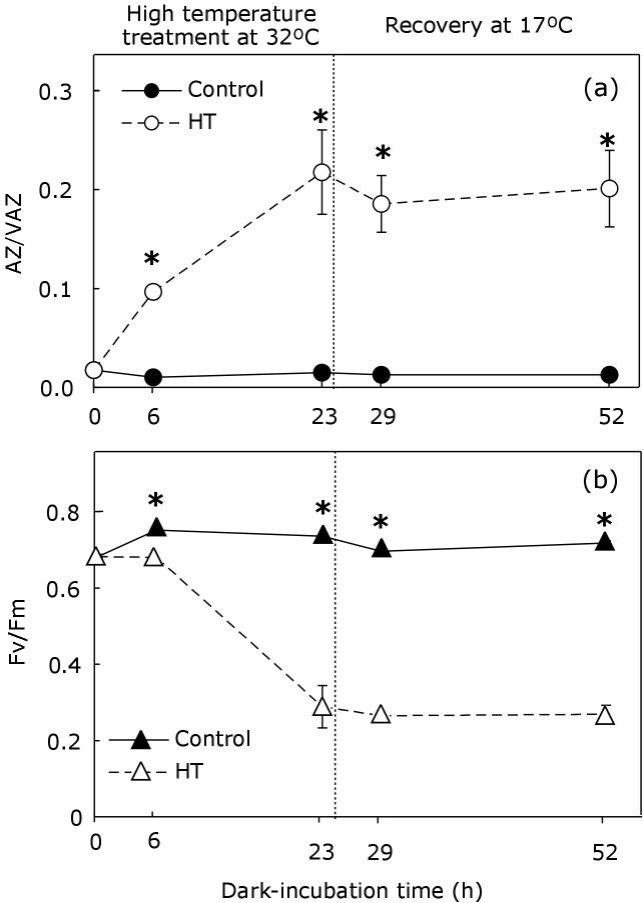
**De-epoxidation index of V-cycle (**a**) and Fv/Fm variations (**b**) during incubation at 32°C in darkness**. Control samples incubated in darkness at 17°C are represented with closed symbols. Treated samples are shown as open symbols. Each symbol represents the mean ± SE (n = 5). Asterisks show significant differences between control and treatment (*P *< 0.05).

The effect of high temperature on the V-cycle was analyzed in thalli incubated in darkness at 32°C (Figure 4). After 6 h of incubation, heat-treated samples showed a significant increase in the AZ/VAZ ratio, which further increased at 23 h (Figure 4a). In parallel, the Fv/Fm decreased throughout the incubation until it reached values of around 0.2 (Figure 4b). In contrast to that observed for desiccation (Figure 2), the return of treated samples to control conditions (17°C) did not reverse the initial values of AZ/VAZ nor of Fv/Fm.

### V-cycle and anoxia

**Figure 5:**
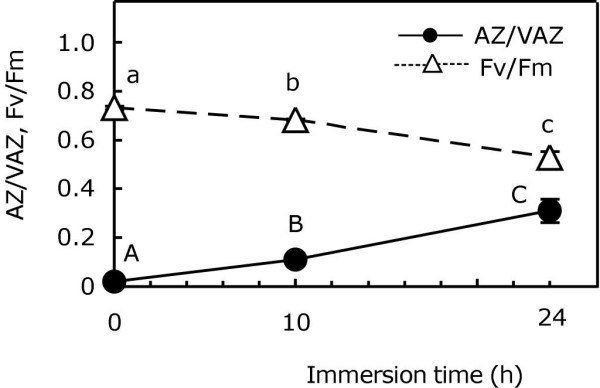
**De-epoxidation index of V-cycle (close circles) and Fv/Fm variations (open triangles) during immersion into seawater in darkness**. Each symbol represents the mean ± SE (n = 5). Letters above bars indicate significant differences for AZ/VAZ (A, B and C) and for Fv/Fm (a, b, c) among immersion times (*P *< 0.05).

Being an intertidal, desiccation-tolerant alga that grows high up on the shore, it has been demonstrated that long, frequent immersions are detrimental for *P. canaliculata*. The activity of the V-cycle was monitored during a long immersion of 24 h in darkness (Figure 5). Surprisingly, despite the absence of light, V was de-epoxidised to A and Z after 10 h of immersion. After disregarding the possibility of any changes in the pH of the water (data not shown) due to respiration, as the factor that induced V-cycle de-epoxidation during immersion, the effects of anoxic conditions were tested separately.

**Figure 6:**
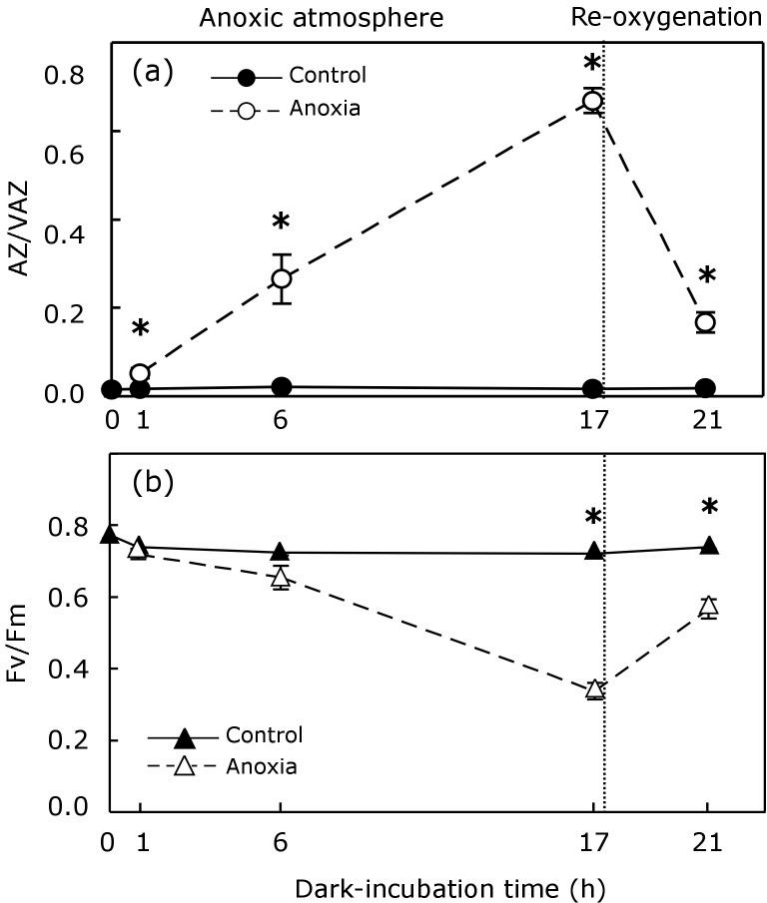
**De-epoxidation index of V-cycle (**a**) and Fv/Fm variations (**b**) during dark-incubation of thalli in a N_2_-atmosphere (anoxic atmosphere) and subsequent recovery in air (re-oxygenation)**. Control samples continuously maintained in oxygenated conditions are represented with closed symbols. Treated samples are shown as open symbols. Each symbol represents the mean ± SE (n = 5). Asterisks show significant differences between control and treatment (*P *< 0.05).

Samples covered with a tissue moistened with seawater were dark-incubated in sealed vials within a N_2_- atm (anoxic atmosphere), while controls were incubated in the same way, but within an air atmosphere. Incubation in anoxia induced the de-epoxidation of a V-cycle (Figure 6a). Under these conditions, one hour was enough to induce a slight, but significant, increase in the AZ/VAZ ratio. After 6 h, it became 12-fold higher than in the controls and after 17 h of incubation, AZ/VAZ increased to values in the range of those reached under high irradiance (compare Figure 6a and Figure 1). In a parallel manner to the increases in AZ/VAZ, Fv/Fm underwent a progressive decrease that after 17 h under a N_2_-atm, was significantly different from what occurred in the control (Figure 6b). After 17 h of incubation, treated samples were transferred to an air atmosphere to allow the re-oxygenation of thalli. After 4 h in an air atmosphere, the de-epoxidation of the V-cycle was reverted, and AZ/VAZ decreased to values below 0.2 (Figure 6a), while Fv/Fm recovered considerably, although it remained below the control value.

### Fv/Fm and V-cycle

**Figure 7:**
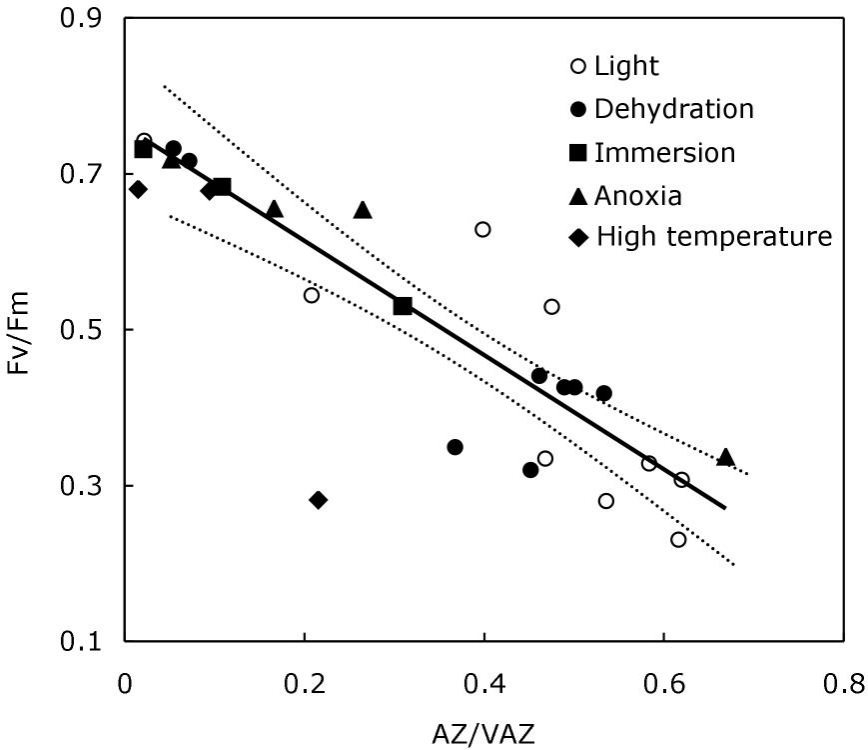
**The relationship of AZ/VAZ and Fv/Fm in *Pelvetia canaliculata***. The data from the different experiments in this work are included: sun light exposition (open circles), dark-dehydration (close circles), dark-immersion (close squares), dark-incubation in anoxia (close triangles), dark-incubation at high temperature (close diamonds). Each point represents the mean (n = 5). The fit (r^2 ^= 0.833) to a linear regression model (solid line) and the 95% confidence intervals (dashed lines) are presented (regression is significant at *P *<0.001). Although showed in the figure, data from the high temperature-treatment were excluded for the regression analysis since it induced irreversible damage on thalli.

The relationship between AZ/VAZ and Fv/Fm was tested for all treatments (high light, dark-dehydration, dark-immersion, dark-anoxia and dark-incubation at high temperature) to verify whether A + Z formed in darkness fits the same regression as that shown in the Figure 1 inset or not and to elucidate if, consequently, this A + Z plays the same role as A + Z formed under light. As shown in Figure 7, irrespective of the treatment, the de-epoxidation of V was highly correlated with Fv/Fm in thalli of *P. canaliculata *(Pearson's correlation coefficient; *r *= 0.906; and *r *= 0.855 when data from the high-temperature treatment were included). In addition, there was a strong relationship between AZ/VAZ and Fv/Fm (Figure 7; linear regression model, *r*^2 ^= 0.833, *P *< 0.001; and *r*^2 ^= 0.731, *P *< 0.01 when data from the high temperature treatment were included).

### Unravelling the common mechanism of V-cycle activation in darkness

**Figure 8:**
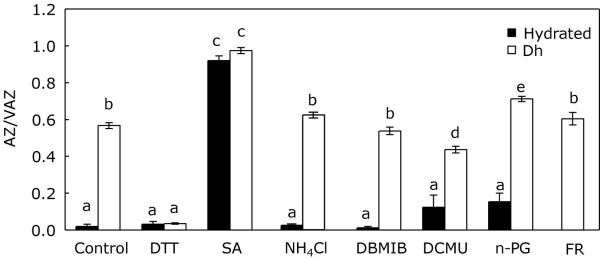
**Effects of V-cycle inhibitors (DTT and SA), transthylakoidal-ΔpH uncoupler (NH_4_Cl) and agents involved in the redox state of PQ (DBMIB, DCMU, n-PG, FR) on the de-epoxidation of V during dehydration in darkness**. Hydrated samples incubated at 100% RH are represented by close bars. The AZ/VAZ after dehydration in darkness is represented by open bars. Each bar represents the mean ± SE (n = 5). Letters above bars indicate significant differences for AZ/VAZ among treatments (*P *< 0.05).

Trying to look more deeply into the biochemical mechanism that triggers the de-epoxidation of V in darkness upon such different stresses, three groups of inhibitors were employed. The first (DTT and SA) affected V-cycle enzymes, the second (NH_4_Cl) uncoupled the transthylakoidal-ΔpH and the third (n-PG, DCMU, DBMIB and FR) altered the redox state of the plastoquinone-pool (PQ-pool). The effect of these inhibitors on the AZ/VAZ was tested in thalli of *P. canalicualta *desiccated in darkness. The DTT brought about a complete inhibition of Z formation during desiccation (Figure 8). The SA, by contrast, led to a strong increase in Z accumulation in darkness. Although after desiccation, the SA-treated samples showed a AZ/VAZ level 30% higher than in the controls (Figure 8), the effect of SA was specially noticeable in hydrated samples in which the AZ/VAZ resulted more than 200-fold higher than in hydrated controls (0.921 ± 0.026 compared to 0.020 ± 0.011). Contrasting with this, the uncoupling of the transthylakoidal-ΔpH by NH_4_Cl did not induce any significant change on the AZ/VAZ (Figure 8).

The third group of inhibitors, acting over the redox state of the PQ pool, had no effect on the hydrated samples and little or no effect on the AZ/VAZ level reached after desiccation (Figure 8). Specifically, when compared with desiccated controls far red (FR) and DBMIB had no effect, DCMU induced a slight decrease and n-PG induced a slight increase on the AZ/VAZ level reached after dehydration.

## Discussion

Photosynthetic organisms living in the intertidal zone need to be able to withstand sudden changes in their environmental factors (such as temperature, salinity, light and desiccation) associated with alternating periods of emergence and submergence. In fact, on rocky shores, seaweed species grow in differentiated horizontal belts characterised by specific levels of light and temperature stresses [29]. Light, influenced not only by the daily course of solar irradiance but also by the tidal range, is one of the most variable and limiting factors for intertidal species. Consequently, seaweeds have developed a set of photoprotective mechanisms that allow them to adjust the amount of light absorbed by their photosynthetic apparatus, thus optimizing its use when light limits photosynthesis, and developing fast photoprotective responses under the effect of photoinhibitory light intensities. Algae possess different photoprotective mechanisms, such as the adjustment of chloroplast orientation [30]; repair mechanisms [31]; the accumulation of UV-absorbing phenolic compounds [32]; or the xanthophyll cycles-related dissipation of the excess of absorbed light energy. The last of these mechanisms is the more flexible and rapid, being crucial for the prevention of photoinhibitory damage. In *P. canaliculata*, the V-cycle pool size is considerably higher than in other brown algae, as corresponds to its position in the uppermost intertidal belt [20]. In addition, it has been demonstrated that this species develops high, non-photochemical quenching (NPQ) when exposed to high irradiance [20]. As shown in Figure 1, light-induced de-epoxidation reverted after the onset of darkness, confirming the complete operation of the V-cycle in this species.

García-Mendoza and Colombo-Pallota [13], have recently described that the brown alga *Macrocystis pyrifera *lacks the initial quenching of fluorescence shown for vascular plants [2], which is associated with the transthylakoidal-ΔpH. As a result, this species shows a direct and linear relationship between the V-cycle de-epoxidation ratio and the NPQ, which does not occur in vascular plants in which the initial NPQ component (qE) is developed due to the ΔpH, independently of V-cycle de-epoxidation. Similarly, a linear negative correlation was found between AZ/VAZ and Fv/Fm (Figure 1, inset), which implies a decrease in photochemical efficiency, mainly dependent on the V-cycle operation.

As previously demonstrated for other phototrophs, such as ferns and chlorolichens [11,12], in *P. canaliculata *the V-cycle operation can be induced solely by dehydration, independently of light (Figure 2). Furthermore, dehydration triggered the de-epoxidation of V and the down-regulation of Fv/Fm to the same extent as illumination did (Figures 1 and 2). To clarify the similarities between light- and dehydration-induced V-cycle activities, a separate experiment was performed in which each branch of the same thalli were either exposed to light or kept in darkness, and subsequently dehydrated in darkness (thereby generating a branch in which A + Z formation was triggered by light and another branch in which this formation was induced by dehydration). As shown in Figure 3, Fv/Fm decreased to the same extent irrespective of the origin of V de-epoxidation, suggesting that A + Z play the same regulatory role when their formation is induced either by light or by dehydration.

Emersion frequently favours overheating, which damages temperate algae [18]. When *Pelvetia *thalli were exposed to 32°C, the heat treatment induced an increase in A + Z but the AZ/VAZ reached was lower than that reached after any other treatment, i.e., dehydration (Figure 2), immersion (Figure 5), or anoxia (Figure 6). The decrease in Fv/Fm was notably larger than that observed for the same level of de-epoxidation in other treatments (note the outlayer point in Figure 7). Besides, the decrease in Fv/Fm and the increase in AZ/VAZ were not reversible after treatment had ceased, indicating that the stress was probably beyond the lethal threshold for a cool water macroalga. Indeed, for other species it has been recently shown that moderate heat stress can affect thylakoid reactions greatly [33].

Besides desiccation and high temperature, periodic hypoxic episodes in intertidal pools may be detrimental for the photosynthetic organisms of those habitats [34,35], especially at night (when no photosynthetic production of oxygen can counteract respiration). Astonishingly, immersion (Figure 5) or anoxia followed by re-oxygenation in an absence of light (Figure 6), induced the same effects on the V-cycle and Fv/Fm as did dehydration-rehydration or light-darkness cycles. The activation of VDE by immersion may be due to the cellular acidification induced by the release of respiratory CO_2_, or by fermentative metabolism [36], but this hypothesis seems unlikely because of the intrathylakoidal location of this enzyme.

Considering the unlikelihood of that hypothesis, the acidification of lumen due to chlororespiration would become a mechanism to take into account. Essentially, during chlororespiration, the plastid terminal oxidase (PTOX) may oxidize plastoquinol (PQH_2_) by transferring electrons to oxygen [37] and at the same time, the pumping of protons towards the lumen would take place [38], providing the lumenal acidic pH required for the VDE activation. Several authors have described the activation of the chlororespiratory pathway by anoxia [39,40]. Although the accumulation of de-epoxidized xanthophylls under chlororespiratory conditions was never observed in green algae [41], chlororespiration has been recently proposed as the mechanism responsible for the dark activation of the diadinoxanthin cycle in diatoms under anoxic conditions [40]. Apart from anoxia, Brüggemann and co-workers have recently proposed chlororespiration as the mechanism responsible for the AZ/VAZ increase observed during the dark incubation of some winter-acclimated oaks at room temperature [10]. Both these examples suggest that chlororespiration may represent a plausible explanation for the activation of V de-epoxidation showed by *P. canaliculata *in darkness. This may be the case of desiccating or overheated tissues, but under anoxia, it is unlikely that PTOX would be able to oxidize PQH_2 _due to the absence of the electron acceptor (oxygen), even when it is considered that under these conditions the PQ pool should be over-reduced [42]. Furthermore, the inhibition of PTOX by n-PG did not block the de-epoxidation of V-cycle pigments that occurred in darkness during desiccation (Figure 8). This result indicates that another mechanism, other than the PTOX-mediated oxidation of PQ, may be responsible for the generation of the transthylakoidal-ΔpH needed for the VDE activation. Nevertheless, the fact that Z accumulation during desiccation of samples in darkness was unaltered by the application of the uncoupler NH_4_Cl (Figure 8) led us to consider an alternative explanation for the de-epoxidation of V in darkness.

It is sometimes thought that under strong light conditions only VDE is activated whereas only ZE works at night or under low light conditions. Nevertheless, ZE activity seems to be constitutive [43]. The complete de-epoxidation of V induced in darkness by SA (Figure 8), together with the inhibition of V de-epoxidation induced by DTT, suggests that also VDE activity is constitutive, even in darkness. In the absence of light, Z accumulation is not observed under non-stressful conditions because ZE prevents its accumulation. However, the inhibition of ZE induced by stress would lead to the de-epoxidation of most of V, and to the consequent increase in the protective Z. Since ZE requires molecular oxygen as second substrate an NADPH as cofactor, both molecules may limit the ZE activity [43]. The underlying mechanism responsible for such inhibition is unknown, but it may be associated with the availability of NADPH, as has been shown in *Arabidopsis *mutants lacking a chloroplastic NAD Kinase [9]. In these mutants, ZE reduces its activity due to the reduced availability of its cofactor NADPH. Consequently, these plants accumulate high amounts of Z even in low light [9].

## Conclusions

In this study it has been shown that, irrespective of the nature of the factor inducing V de-epoxidation, resultant A + Z molecules always contribute to the down-regulation of photochemical efficiency. In fact, when AZ/VAZ values against Fv/Fm for all the experiments reported here were plotted in the same regression, a significant linear dependence was observed between them, irrespective of the stress factor (Figure 7). This observation provides a physiological meaning for the reported dark activation of the V-cycle, but also opens the question about the role and adaptive values of this trait. All the experimental evidence [4] supports the theory that VDE activation requires an acidic pH, which is typically generated by light-induced proton pumping, but the dark accumulation of Z requires a different explanation, probably associated with the down-regulation of ZE activity. Although the molecular basis of ZE down-regulation remains unclear, there is some evidence that phosphorylation reactions might be involved in this [22,44,45].

Considering the emerging number of stressors that lead to Z formation in darkness, and the multiple protective roles of this xanthophyll in membrane stabilization [46,47], in the prevention of the reactive oxygen species (ROS) generation [48,49] and in the scavenging of ROS [50], it seems that, more than being a biochemical curiosity, V de-epoxidation in darkness must represent a very important and finely-regulated protective mechanism. The wide occurrence of V-cycle activity in darkness in terms of phylogenetic groups [51] and in terms of inducing factors (as shown in this work) may imply that this is a common response to most stressors that has been conserved tenaciously during the evolution, to protect thylakoid membranes and photosynthetic machinery under hazardous conditions even when these occur in the dark.

## Abbreviations

A: antheraxanthin; DBMIB: Dibromo isopropyl methyl benzoquinone; DCMU: Dichlorophenyl dimethylurea; DTT: dithiothreitol; Fv/Fm: maximal photochemical efficiency of the PS II; HT: high temperature; NPQ: non-photochemical quenching; n-PG: n-propyl-gallate; PTOX: plastid terminal oxidase; R: rehydration; RH: relative humidity; SA: salicyl-adoxime; V: violaxanthin; V-cycle: violaxanthin cycle; VDE: Violaxanthin de-epoxidase; Z: zeaxanthin.

## Authors' contributions

BFM and JIGP designed and performed the experiments. FM and BFM carried out the HPLC analyses. JIGP, BFM and FM analyzed fluorescence. JMB BFM and JIGP performed the experiments with inhibitors and the statistical analysis. All authors read and approved the final version of the manuscript.
